# Co-Regulation of Histone-Modifying Enzymes in Cancer

**DOI:** 10.1371/journal.pone.0024023

**Published:** 2011-08-23

**Authors:** Abul B. M. M. K. Islam, William F. Richter, Laura A. Jacobs, Nuria Lopez-Bigas, Elizaveta V. Benevolenskaya

**Affiliations:** 1 Department of Biochemistry and Molecular Genetics, University of Illinois at Chicago, Chicago, Illinois, United States of America; 2 Research Unit on Biomedical Informatics, Department of Experimental Health and Sciences, PRBB, Universitat Pompeu Fabra, Barcelona, Spain; Roswell Park Cancer Institute, United States of America

## Abstract

Cancer is characterized by aberrant patterns of expression of multiple genes. These major shifts in gene expression are believed to be due to not only genetic but also epigenetic changes. The epigenetic changes are communicated through chemical modifications, including histone modifications. However, it is unclear whether the binding of histone-modifying proteins to genomic regions and the placing of histone modifications efficiently discriminates corresponding genes from the rest of the genes in the human genome. We performed gene expression analysis of histone demethylases (HDMs) and histone methyltransferases (HMTs), their target genes and genes with relevant histone modifications in normal and tumor tissues. Surprisingly, this analysis revealed the existence of correlations in the expression levels of different HDMs and HMTs. The observed *HDM*/*HMT* gene expression signature was specific to particular normal and cancer cell types and highly correlated with target gene expression and the expression of genes with histone modifications. Notably, we observed that trimethylation at lysine 4 and lysine 27 separated preferentially expressed and underexpressed genes, which was strikingly different in cancer cells compared to normal cells. We conclude that changes in coordinated regulation of enzymes executing histone modifications may underlie global epigenetic changes occurring in cancer.

## Introduction

The genome of any multicellular organism contains thousands of genes; however, only a relatively small proportion of these genes are expressed in a particular cell type. Part of the critical functional characteristics between active and repressed states of gene expression relates to proteins that bind to and modify histone lysine residues. Histone lysine methylation occurs predominantly within the amino-terminal tails of histones H3 and H4, in a mono- (me1), di- (me2) or trimethylation (me3) state, and is associated with a distinct transcriptional outcome. These diverse methylated residues are bound by multiple “reader” proteins, which in turn interact with transcriptional activators or repressors. H3K4me2/3, H3K36me1/3, H3K79me1/2 and H4K20me1 are associated with transcriptional activation, while H3K9me2/3, H3K27me2/3, H3K79me3 and H4K20me3 are associated with transcriptional repression. With the recent discovery of histone demethylases, modification of histone lysine residues is now viewed as a more dynamic process than previously thought. Multiple studies have shown that histone-modifying enzymes target specific genes, some of which participate in specific biological processes and pathways. It is still under debate whether targets of a histone-modifying enzyme achieve regulation as a single module, allowing coordinated change in expression in various biological processes, including diseased conditions, or represent a mix of differentially expressed genes.

Histone demethylases are represented by a few flavin-dependent amine oxidases and α-ketoglutarate-Fe(II)-dependent dioxygenases that are included in a large superfamily of the JmjC domain proteins (Table S1). Histone methylation is also accomplished by multiple enzymes, and in most cases involves a catalytic SET domain (Table S1) related to yeast Set1 and *Drosophila* Trithorax. The same modification can be executed by several enzymes, which in most cases are members of the same protein family, suggesting their functional specialization through differential expression patterns. Histone demethylases removing the methyl groups from histone H3K4 are encoded by two autosomal genes, *KDM5A/JARID1A/RBP2* and *KDM5B/JARID1B/PLU1*, and two genes located on the sex chromosomes, *KDM5C* and *KDM5D*, and increasing evidence supports individual and nonredundant roles within this family [1]. Therefore, it is conceivable that the overall epigenetic landscape is dependent on the spatial and temporal distribution of corresponding HDM and HMT enzymes. KDM5A is directly bound to H3K4me3 in vivo [2]. Despite the repressive demethylase activity associated with KDM5A function in demethylation at histone H3K4, it plays a role in both transcriptional repression and activation [3]. KDM5A contains not only an enzymatic domain but also a highly specific H3K4me3 reader domain [4], which without doubt could affect other modifications nearby either cooperatively or antagonistically. Consistent with these observations, our ChIP-on-chip analysis showed that KDM5A binding to genomic loci highly correlates with transcriptionally active promoters containing H3K4me3 and other modifications associated with transcriptional activation, such as H3K36me3, H3K79me2 and acetylation at H3K9 and K14, but not H3K27me3 [5]. Considering the diverse functions identified for families of enzymes like KDM5, it will be of particular interest to understand their contribution to histone H3K4 methylation in promoter- and cell type-dependent manner.

Genomic analyses in *Drosophila* and mammals showed that Trithorax group (TrxG) genes antagonize regulation by Polycomb group (PcG) genes to create a repertoire of alternative states of gene expression. Accumulating evidence has shown that recruitment of HMTs or HDMs of opposing activity to the same genomic regions underlies important developmental decisions. In particular, the HMTs MLL1 and EZH2 are components of the Trithorax and Polycomb complexes, respectively, that play an important role in the regulation of developmental genes in both *Drosophila* and vertebrates. Based on the enhancement of phenotype of trxG mutations and suppression of phenotype of PcG mutations, the *KDM5A* ortolog in Drosophila, gene *lid* belongs to the trxG group gene [6]. The effects of mutations in the PcG and TrxG genes in *Drosophila* led to the paradigmatic view of homeotic (*HOX*) gene regulation. In vertebrates, *HOX* genes are also sensitive to loss of the H3K4-specific HMT MLL1 or HDM KDM5A, and H3K27-specific HMT EZH2 [4], [7], [8], [9], [10]. As in *Drosophila*, deletion of the MLL1 SET domain in mice results in a homeotic phenotype [11]. Importantly, rearrangements of the *MLL1* gene in humans are involved in the pathogenesis of a variety of aggressive human leukemias. The disregulation of *HOX* genes by MLL1 fusion proteins appears to play a central role in this transformation [9]. Following elucidation of the role of MLL translocations in the pathogenesis of leukemia came the discovery of a KDM5A leukemia oncogenic lesion, resulting in the deregulation of *HOX* and other lineage-specific genes [4]. Considering the requirement for the proper level of expression for each of these enzymes involved in HOX gene regulation, it is conceivable that each state is the result of a combined activity of histone modifying enzymes. A recent study suggested that this explains dynamics of each state, when switching to a different state is dependent on the relative levels of PcG, TrxG and activators in *cis*-regions of the locus [12]. In particular, differentiation of stem cells involves the resolution of the poised state of “pluripotency genes” and lineage-specific genes through a change in the balance of PcG and TrxG complexes. These genes were found to be enriched for bivalent H3K4me3 and H3K27me3 marks. In contrast, cells can regain stem-cell characteristics through reactivation of pluripotency genes and repression of the lineage-specific program, which was associated with the resolution of bivalent mark into the corresponding univalent mark. The ability of cells to maintain the poised, repressed or fully active states of these genes [12] is critical for progression to a cancerous state. It has been hypothesized that underlying these events is a change in the TrxG/PcG balance in favor of a particular complex [13].

It is somewhat surprising that, despite the large amount of newly generated gene-expression data in normal or tumor tissues, there were no expression studies of targets of histone modifying enzymes at the global level. Using representative, internally consistent expression datasets from a variety of normal and tumor tissues and from cell lines, we have identified cell types and tumors where the genes encoding HDMs and HMTs are prominently expressed and underexpressed. Previous studies have shown dependence of expression of particular HDM/HMT target genes on the level of a HDM or HMT. Here we analyzed large sets of epigenetically regulated genes, including a set of direct targets of KDM5A, a set of direct targets of EZH2, and sets of H3K4 and H3K27 trimethylated genes. Strikingly, our analysis revealed that the whole set of genes can highly correlate in expression with the level of gene expression of the corresponding enzyme. Consistent with opposing activities of TrxG and PcG proteins, this analysis revealed anti-correlation in the expression of KDM5A and EZH2 targets, and of genes characterized by relevant histone modifications. This study enabled identification of sets of co-regulated *HDMs* and *HMTs*, comprising a *HDM*/*HMT* gene expression signature. The correlations that we observed in normal tissues were different from correlations identified in cancer cells. Considering multiple alterations in HDM and HMT genes in cancer cells, it seems that using this approach could yield new functional connections between histone modifying enzymes, which might be helpful in designing a combination cancer therapy.

## Results

### Correlations in expression of HDMs, HMTs, their targets and genes with histone H3K4 and H3K27 methylation

The most widely studied marks of active and repressed chromatin are on histone H3 methylated at K4 and at K27, respectively. KDM5A significantly overlaps with the H3K4me3 marks at the regions around the transcription start sites, and EZH2 binds throughout H3K27me3 regions [14]. We previously showed that consistent with the presence of H3K4me3 mark at the genes occupied by KDM5A in U937 cells, these regions were highly transcriptionally active in U937 cells [5]. We also described, using *z*-score analysis, preferential expression of this set of KDM5A targets in human tissues [5]. This suggested that KDM5A target genes form a module characterized by higher expression and presence of H3K4me3. While it is generally assumed that both features are dependent on presence of enzymes involved in H3K4 methylation, the correlation between the level of expression of this module and enzyme in the same cells has not been demonstrated.

**Figure 1:**
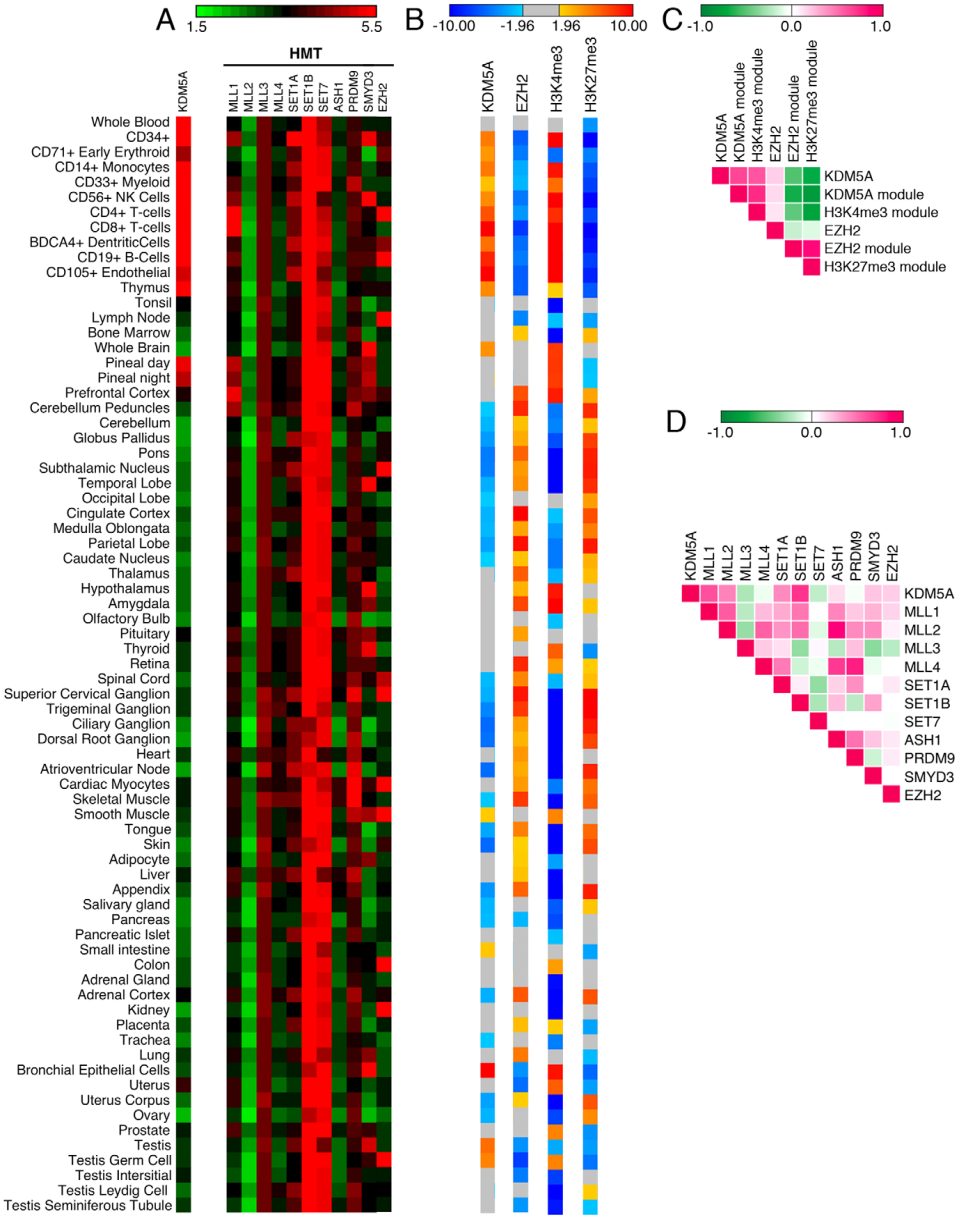
Expression and correlation of H3K4 modifying enzymes and their targets in normal human tissues. (A) The absolute (log_2_) expression values of H3K4-specific HDM KDM5A, and H3K4-specific HMTs in 73 different normal human tissues (BioGPS database) are delineated in a color-coded heatmap, where red indicates higher expression of a gene and green indicates lower expression. The expression values and sample annotation are presented in Table S2. Several genes, including *KDM5A* and *MLL1*, display higher expression in a small set of cell types. (B) *Z*-score values for preferential expression and underexpression of target genes of KDM5A (U937 cells), EZH2 (ES cells) and genes displaying H3K4me3 or H3K27me3 (MCF-7 cells). *Z*-score values are delineated in a different colored heatmap, where red signifies overexpression of targets and blue signifies underexpression of targets; grey indicates no significant difference from expected value. (C) Pearson correlation coefficient (PCC) of the expression level of *KDM5A*, *EZH2* and of gene modules in (B). Expression of KDM5A module and of H3K4me3 module correlates with expression of *KDM5A* gene. Strikingly, expression of both modules displays negative correlation with the expression of EZH2 module and H3K27me3 module. (D) PCC of the expression profile of any pair of genes in (A). A high correlation is displayed between *KDM5A* and the *MLL1* gene expression, which may account for the general correlation of *KDM5A* and its targets in (C). PCC values are depicted in a color-coded heatmap using a standard 1 bases scale, where magenta (+1) shows higher positive correlation and green (−1) shows higher anti-correlation. The PCC values for (C) and (D) are provided in Tables S4 and S5.

Given the differential expression of KDM5A module in particular tissues, we examined if *KDM5A* is also differentially expressed in the same tissues. Indeed, analysis of *KDM5A* normalized expression data from the compendium of human tissue samples in GeneAtlas (BioGPS database) showed that *KDM5A* is highly expressed in cells derived from bone marrow and peripheral blood, where its target genes are preferentially expressed (*z*-score >1.96) (Figure 1A and B, Table S2 and Table S3). Using Pearson's correlation coefficient (PCC) to describe the correlation in expression of KDM5A module with the level of *KDM5A*, we found that the correlation was very significant across all tissues (PCC = 0.65 in Figure 1C and Table S4).

One interesting question is to compare expression of targets of a HDM/HMT with general expression of genes with the relevant histone modification. Given the direct binding of KDM5A to H3K4me3 [2], we analyzed expression of genes displaying H3K4me3 and compared it to the expression of KDM5A targets. Previously we showed that if we take in our analysis diffuse histiocytic lymphoma U937 cells or U937 cells differentiated in monocytes and macrophages, despite different sets of KDM5A targets, the analysis shows their preferential expression in the same set of tissues [5]. Therefore, in subsequent analyses we deliberately used gene targets obtained from different cell lines. We continued using KDM5A dataset from U937 cells but used H3K4me3 dataset from breast adenocarcinoma MCF7 cells [15]. Using these datasets, we found that tissues with overexpression of *KDM5A* and KDM5A target genes also display preferential expression of genes displaying H3K4me3. Comparison across the GeneAtlas tissues showed that the genes with H3K4me3 were preferentially expressed in the same tissues where KDM5A target genes were overexpressed (PCC = 0.74), suggesting that the overexpression of KDM5A target genes is due to H3K4 methylation.

As H3K4 HMTs could directly affect expression of targets of the HDM KDM5A, we asked whether we could identify an H3K4 HMT whose level correlated with *KDM5A* expression level, and may account for higher expression of H3K4me3 module in GeneAtlas. We observed relatively uniform expression for *MLL2*, *MLL3*, *SET1B* and *SET7* across different tissue samples (Figure 1A and Table S2). In contrast, *MLL1*, *SET1A*, *PRDM9* and *SMYD3* demonstrated highly tissue-specific patterns of expression. *SMYD3* displayed prominent expression in the central nervous system. There was higher expression of *MLL1* in hematopoietic cells, especially in CD4^+^ and CD8^+^ T-cells, displaying preferential expression of H3K4me3 module. Consistent with these observations, mouse CD4^+^CD8^+^ T-cells are known to express the *MLL1* gene [16] and H3K4 methylation correlates highly with gene activation in CD4^+^ T cells [17]. Using PCC to describe the correlation in expression level in different gene pairs, we found that *KDM5A* and *MLL1* displayed high correlation of expression of (PCC = 0.58 in Figure 1D and Table S5). As well as in blood cells, *MLL1* and *KDM5A* show high expression levels in the pineal gland, and this correlated with the higher expression of H3K4me3 module. The pineal gland establishes a circadian rhythm for the circulating hormone melatonin. Several histone modifications associated with activated transcription have been shown to oscillate in the pineal gland [18]. *MLL1* and *KDM5A* may contribute to differential transcription in the pineal gland, which may be as high as 100-fold, through oscillation of H3K4me3. These data suggest that multiple H3K4 HMTs exhibit highly tissue-specific patterns of expression. There is a consistent pattern of tissue specificity in the expression of MLL1 and KDM5A, two histone-modifying enzymes involved in leukemia. Their levels might be adjusting to fulfill requirements in H3K4-mediated processes.

The above result was consistent with a significant overlap between KDM5A targets and H3K4me3 regions in multiple cell lines [14]. To examine if the same true for targets of other histone modifying enzymes, we analyzed expression of genes with H3K27me3 from the same study in MCF7 cells and of EZH2 target genes determined in ES cells [19]. Again, we found high correlation in the level of expression of both modules (Figure 1B and 1C). Unexpectingly, in the same tissues where KDM5A targets were preferentially expressed, the EZH2 targets were significantly underexpressed (PCC = -0.68 in Figure 1C and Table S4), which highly correlated with underexpression of genes displaying H3K27 methylation (PCC = −0.82). Target genes determined for EZH2 were underexpressed in cells derived from bone marrow and peripheral blood (*z*-score <−1.96) (Figure 1B). Strikingly, in differentiated tissues such as brain the gene expression states were the opposite, with overexpression of the EZH2 module and underexpression of the KDM5A module. However, the anti-correlative behavior between the level of expression of both modules remained in all tissues. These data indicate on the existence of an intimate crosstalk in establishing expression levels of KDM5A module and EZH2 module. This represents an important observation suggesting that epigenetic landscape can be described as a simple combination of modules dependent on HDM/HMT expression.

### *HDM* and *HMT* tissue-specific expression profiles

**Figure 2:**
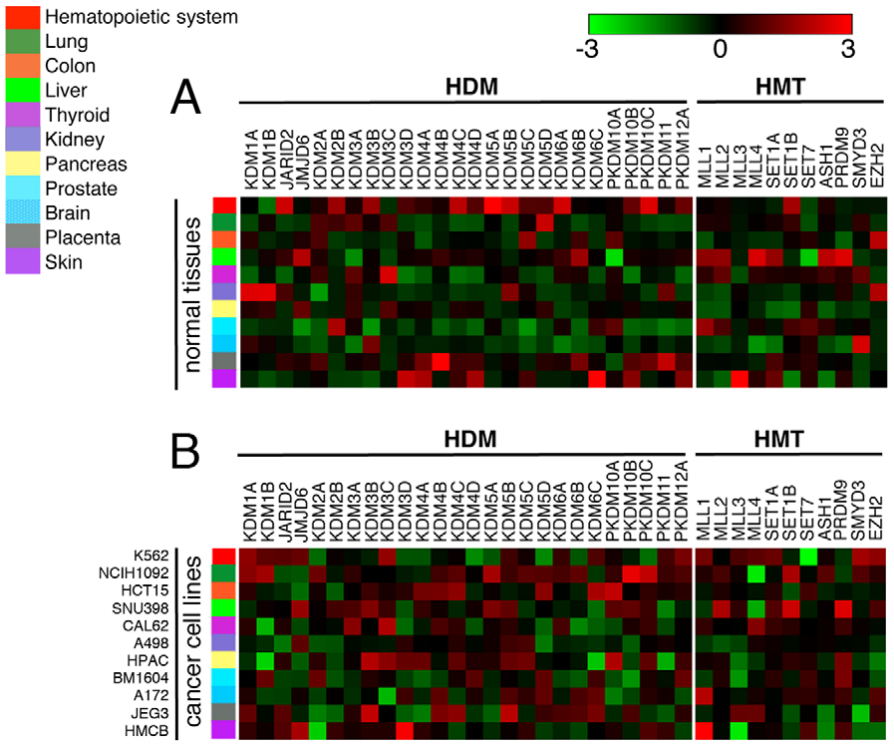
Expression of *HDM*s and *HMT*s in normal human tissues and human cancer cell lines. (A) Expression of *HDM*s and *HMT*s in normal human tissues. Gene-normalized expression values (log_2_) of 26 HDMs and 11 HMTs are presented for eleven tissues (BioGPS database). (B) Expression of *HDM*s and *HMT*s in cancer cell lines. Gene-normalized (log_2_) expression values were generated for eleven cell lines from GSK dataset. Expression level for each gene was described in terms of preferential expression and underexpression, which showed relative higher expression of multiple *HDM* and *HMT* genes in cancer cells compared to normal cells.

Within *JmjC* genes, 12 gene families can be defined on the basis of phylogenetic information and overall domain structure [20]. Many of these families have experienced birth-and-death evolution, including duplication in one lineage and loss in another lineage. The *KDM5* subfamily, where different gene members have highly specialized functions, emerged from gene duplication events after the divergence of vertebrates from insects. Indeed, different *KDM5* family genes display tissue-specific expression, reflected not only in sex-specific expression but also in autosomal expression (Figure 2A). In addition, some tissues may depend less on histone demethylases than other tissues, and methylation may instead be removed by other mechanisms. To address this, we asked whether there were tissues expressing *HMTs* with an undetectable or low level of *HDMs*. We found that the prostate was relatively deficient in *HDMs* while showing relative higher expression of *HMTs* (Figure 2A). An increase in HMT activity could potentially lead to histone hypermethylation in prostate carcinoma patients. In contrast, preferential expression of *HDMs* was observed in the hematopoietic system, while *HMTs* were highly expressed in the liver. Surprisingly, the brain was relatively deficient in both *HDMs* and *HMTs*. To find if the *HDM/HMT* expression pattern stays the same during neeoplastic transformation, as the first step, we performed similar analysis from cancer cell lines from GSK dataset. The expression pattern was drastically different in cancer cell lines compared with cells from normal tissue (Figure 2A and 2B). While *HDMs* were generally underexpressed in the prostate and brain tissues of healthy individuals (Figure 2A), particular HDMs were found to be overexpressed in prostate and brain cancer-derived cells (Figure 2B).

### Cancer type-specific gene expression changes in HDMs/HMTs and their targets

**Figure 3:**
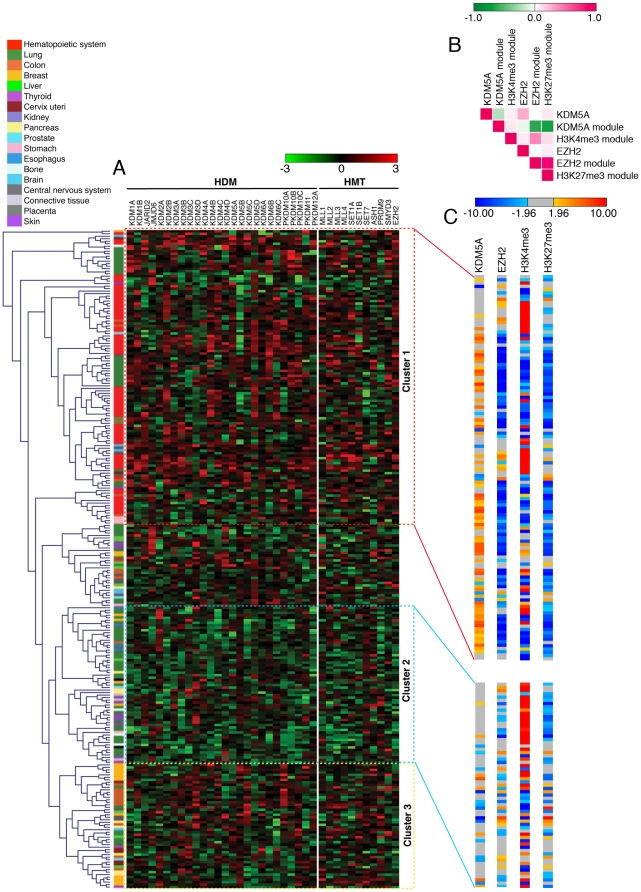
Clustering of cancer cell lines based on *HDM* and *HMT* gene expression, or on the expression of their targets. (A) Unsupervised hierarchical clustering of 264 different cancer cell lines of 18 tissue types (GSK database) for the expression of *HDM*s (left panel) shows three major clusters. Log_2_ normalized gene expression is represented by a color-coded heatmap. Red indicates relative higher expression and green indicates relative lower expression from the gene's mean of all cell lines. Columns represent genes and rows represent samples. The normalized expression of different *HMT*s is presented in the right panel. The legend and scale are as in Figure 2. (B) PCC of the expression level of *KDM5A*, *EZH2* and of gene modules in (C) in cluster 1. PCC values are depicted as in Figure 1. (C) Enrichment analysis, using *z*-score statistics, of the expression level of displayed gene modules as in Figure 1(B). The complete list of *z*-score values for each sample and gene are provided in Table S7. In this dataset, the samples from the same tumor type clustered together on the basis of either HDMs, HMTs or their target gene expression.

Based on the analysis above, we hypothesized that coordinated regulation underlying functional interactions among cooperating and opposing HDM and HMT activities is critical for tumors as well as for normal tissue, but that in tumors we would detect distinct correlations in gene expression. To test this hypothesis, we used the GSK dataset, the largest collection of gene expression data available for cancer cell lines. This set of 264 samples mostly includes hematopoietic and lung cancer-derived cell lines, and varying numbers of cell lines from 16 other tissues (Table S6). We performed unsupervised hierarchical clustering of samples in order to identify an *HDM* gene expression signature in cancer. This analysis revealed three large clusters (Figure 3A). Surprisingly, when the cell lines were arranged according to *HMT* expression, it showed a similar grouping (data not shown), suggesting that expression of *HDMs* and *HMTs* is interdependent. We found that samples within each cluster originated from specific cancers; the lung cancer cell lines were found in all three major clusters but were restricted to subclusters. The main cluster 1 (in the red boxed area) showed relatively high expression and comprised hematopoietic cell lines. Cluster 2 (in the blue boxed area) was formed by skin, pancreas and bone cell lines, which showed relatively low expression. Cluster 3 (in the yellow boxed area) showed intermediate expression, with breast, colon and central nervous system cancer cell lines forming this group.

Several lines of evidence support the accuracy of the revealed *HDM/HMT* gene expression signatures. Consistent with deregulation of KDM5A in leukemia [4], this gene was prevalently expressed in cluster 1, and the *KDM5A* overexpression seen in lung cancer cell lines may be due to their CD133-positive character [21]. Expression of the *KDM5A* homolog *PLU1/KDM5B* was, in contrast, very different from *KDM5A* expression. *KDM5B* was absent from cluster 1 and was very highly expressed in breast carcinoma cell lines in cluster 3. Previous studies showed *KDM5B* overexpression in advanced breast carcinoma and breast cancer cell lines [22]. Consistent with upregulation of EZH2 in aggressive cancers [23], the clustering revealed high expression of *EZH2* in cluster 1, which contained samples from highly aggressive blood and lung cancers.

Because HDM and HMT displayed cancer-specific gene expression signature, we asked whether correlations in their target gene expression or in the expression of genes marked with histone methylation are lost in cancer samples. We acquired expression data for KDM5A targets and EZH2 targets from the GSK dataset and applied *z*-score analysis to study the significance of their combined up- or downregulation in different cancer cell lines samples. We found that similar to normal cells, EZH2 targets are strongly biased towards similar expression with the H3K27me3 module (PCC = 0.86), while KDM5A module displayed anti-correlative behavior to the expression of EZH2 module (PCC = −0.67) and H3K27me3 module (PCC = −0.73) (Figure 3B). Samples with the highest *z*-score values for KDM5A module and with the lowest *z*-score values for EZH2 module were found in leukemia samples of cluster 1 (Figure 3A and Table S7), which was consistent with high expression of these modules in normal blood cell types (Figure 1B). However, in contrast to normal tissues, there was no tendency for the H3K4me3 module to be overexpressed in the same cancer samples as the KDM5A module (PCC = 0.07) (Figure 3B, 3C and Table S8). Even more striking differences were displayed in cluster 2, where most of *HDMs* and *HMTs* were expressed lower. In cluster 2, there were no significant correlations in expression of any of the four modules (Figure 3C). Therefore, cancer-specific *HDM*/*HMT* gene expression signature is likely to dictate global changes in histone methylation affecting target gene expression.

**Figure 4:**
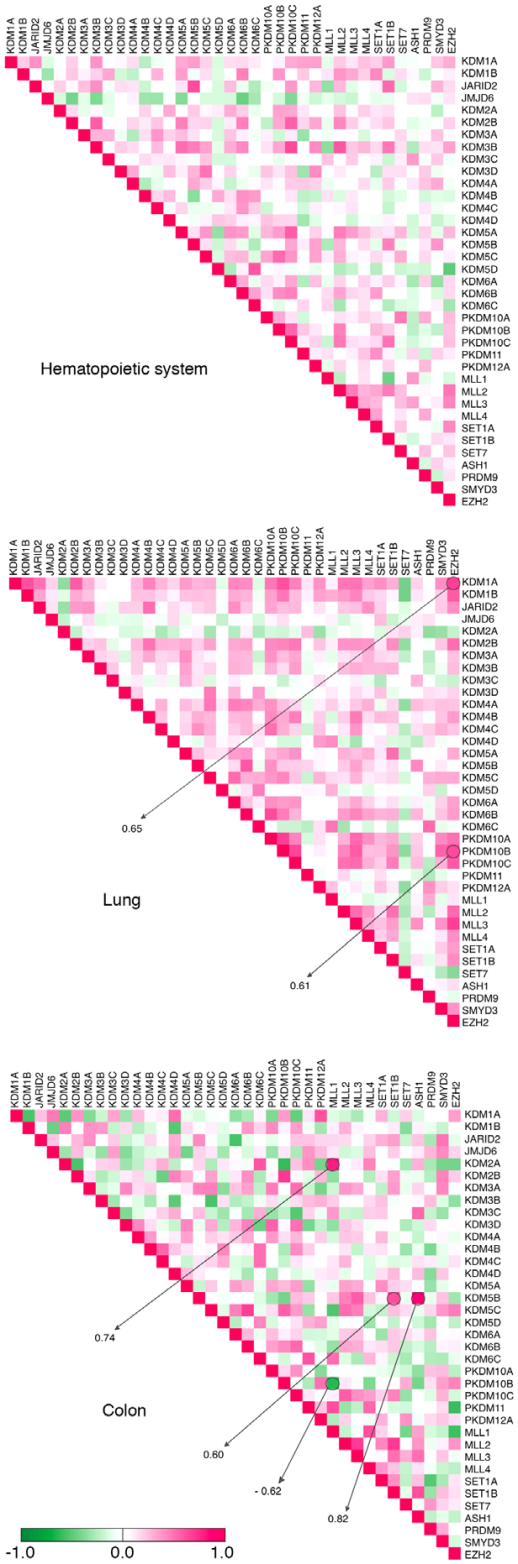
Correlation between *HDM* and *HMT* gene expression. The PCC values of any gene pair's expression profile in hematopoietic, lung and colon cell lines are depicted in a color-coded heatmap. The legend and scale are as in Figure 1.

As the GSK samples from the same tumor types clustered together on the basis of either *HDMs* or *HMTs* gene expression, we asked whether we could reveal any cases of *HDM* and/or *HMT* co-regulation in human cancer. We computed pair-wise associations among *HDMs* and *HMTs* (Table S9), allowing the identification and visualization of “enrichment” of linked pairs (Figure 4). We have shown a high correlation between *KDM5A* and the *MLL1* gene expression, which may account for the general correlation in expression of *KDM5A* and its targets (Figure 1). However, in cancer cells we were unable to detect correlation between *KDM5A* and *MLL1* expression, even when only hematopoietic cell lines were considered (PCC = −0.03). The highest PCC values, including the anti-correlation between *PKDM10B* and *MLL1* (PCC = −0.62), and correlation in *KDM5B*–*ASH1* (PCC = 0.82) and *KDM2A*–*MLL1* (PCC = 0.74) pairs, were revealed in colon cancer cell lines. We found a negative correlation of *EZH2* and *MLL1* expression (PCC = −0.56) in colon cancer and positive correlation of *EZH2* and *KDM1A* expression (PCC = 0.65) in lung cancer, suggesting cooperation between H3K27 methylation and H3K4 demethylation. In many cases, the revealed associations were clustered by protein families. For example, expression of *PKDM10A*, *-B* and *-C* displayed a lung cancer-specific correlation between each other and with other *HDM*s and *HMT*s. Therefore, the new *HDM/HMT* gene expression signature may explain the lack of correlation between the KDM5A module and H3K4 module in cancer (Figure 3B) compared to normal tissue (Figure 1C). The coordinately regulated *HMT*s and *HDM*s may provide functionality in highly specific, yet-to-be-identified, biological processes. When combined with the power of unsupervised hierarchical clustering, PCC analysis may reveal many other correlating pairs.

### Expression of HDMs and HMTs in primary human tumors

**Figure 5:**
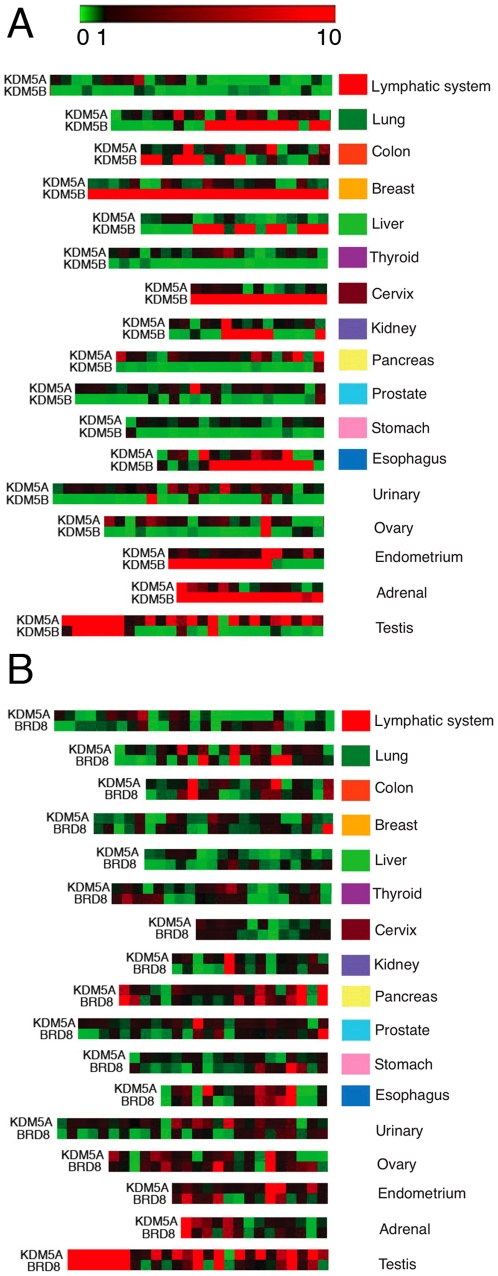
Expression levels of *KDM5A*, *KDM5B* and a KDM5A target gene in human tumors. (A) *KDM5A* and *KDM5B* display highly tissue-specific expression patterns. The figure shows a heatmap of quantitative real-time PCR experiments in a panel of human tumor samples. Samples were arranged according to tumor grade, when information was available. Expression was normalized to the reference gene *B2M* and presented relative to the mean ΔCt of each gene. Heatmaps were produced using Gitools. The scale ranges from the minimum value of 0 (green), indicating low relative expression, to the mid-value of 1 (black) indicating an average level of expression, to the maximum value of 10 (red) indicating high relative expression. Columns represent different patient samples. (B) Expression of the KDM5A target gene *BRD8* provides a readout of *KDM5A* transcript level. Sample annotation and expression values are presented in Table S9.

After this initial assessment of expression patterns in various malignancies using cancer cell line data, we next surveyed transcript levels in hundreds of cancer tissue samples. For these purposes, we used RT-qPCR analysis, which is a better method for quantitation of gene expression than microarray studies. While *KDM5A* and *KDM5B* are a highly homologous pair of genes, their expression patterns in different cell lines varied (Figure 3A). qPCR analysis for *KDM5A* and *KDM5B* levels on a cDNA array containing 337 samples across 17 tissue types showed that the *KDM5A* level was high in the testis and low in lymphoid malignancies (Figure 5A, Figure S1 and Table S10). In contrast, the *KDM5B* level was high in breast tissue, adrenal glands and cervix (Figure 5A). Consistent with *KDM5B* overexpression in malignant breast tumors [22], a highly increase in *KDM5B* level was observed in cDNA samples from primary ductal breast adenocarcinomas (Table S10). *KDM5B* expression also correlated with lung tumor stages, increasing in stages IIB, IIIA and IV. In Figure 1C, we showed that expression of the KDM5A module is predictive of a level of *KDM5A* expression, which is lost in cancer (Figure 3B). While this result is based on calculating median expression value for all genes in KDM5A module, it is unclear whether some genes within the module still correlate with *KDM5A* gene. Yet, due to the importance of KDM5A in human cancer, it is critical to identify a gene that could be used as a readout of KDM5A activity. *BRD8* has been identified as a direct target of KDM5A, positively regulated by KDM5A [3]. In the majority of samples on the cancer tissue array, we found upregulation of *BRD8* when *KDM5A* was upregulated and downregulation when *KDM5A* was downregulated (Figure 5B and Figure S2). However, this was not the case for many other genes, KDM5A targets, that we tested (data not shown). Therefore, *BRD8* expression mirrors *KDM5A* expression, thus validating it as a marker for KDM5A level and activity.

## Discussion

**Figure 6:**
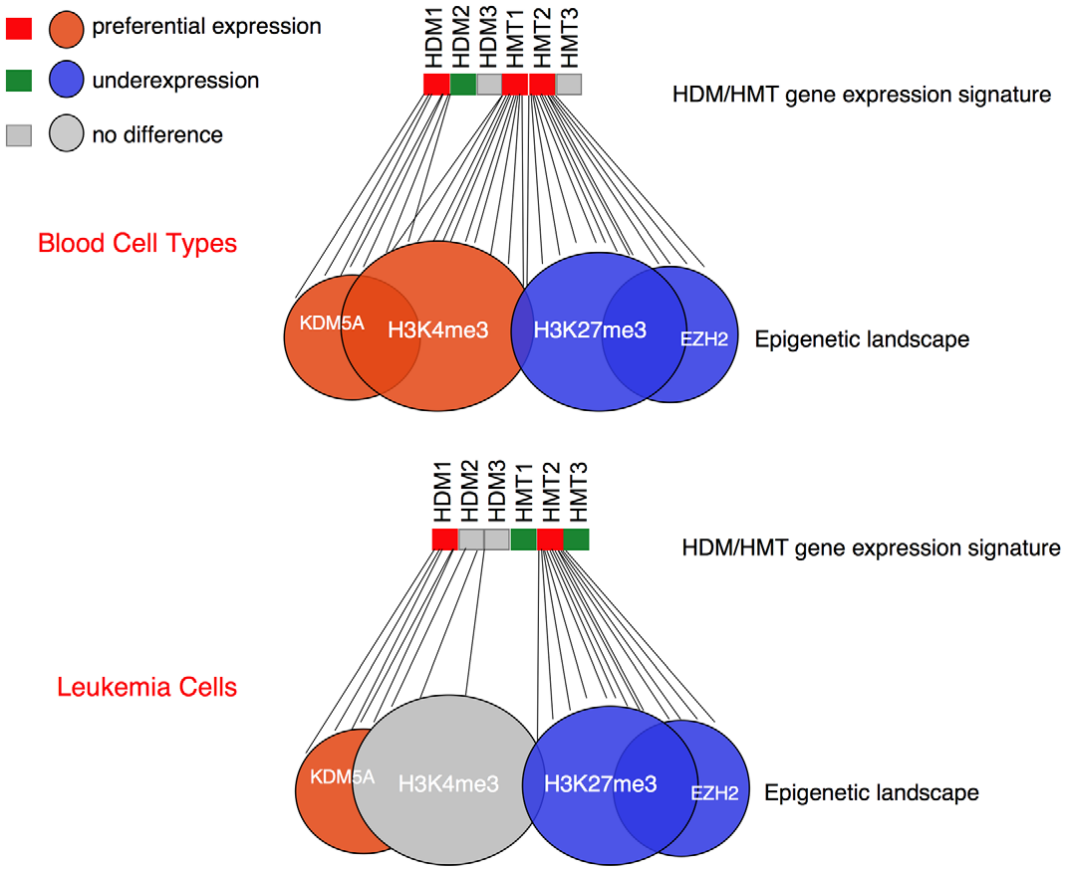
Epigenetic landscape is defined by *HDM*/*HMT* gene expression signature. The status of *HDM* and *HMT* gene expression is presented in red (higher expression) and green (lower expression) colors. The KDM5A, EZH2, H3K4me3 and H3K27me3 modules are shown as large circles, either preferentially expressed (in red color) or underexpressed (in blue color). The expression of KDM5A targets and genes with H3K4me3 is determined by the levels of *HDM1* and *HMT1*, which are relatively high in blood cells. In leukemia cells, the level of *HMT1* becomes lower, the level of *HDM2* becomes higher and as a result, the responsive H3K4me3 module is not preferentially expressed anymore. Some of the H3K4 and H3K27 trimethylated regions overlap as bivalent marks and remain poised for expression (shown as a grey area in the overlap).

Analysis of gene expression in different human tissues and cell lines enabled us to recognize epigenetically regulated genes as single modules that are dependent on the level of expression of histone-modifying enzymes (Figure 6). Much of the work done so has focused on how the binding of these enzymes correlates with the expression of their target loci. In this study, we conducted global analysis of the expression level of the enzymes, their targets and other epigenetically regulated genes. The *HDM*/*HMT* gene expression signatures that we identified are likely to be functionally significant because of the observed correlation with HDM/HMT target gene expression as well as with the expression of genes displaying relevant histone marks. Specifically, there is a strong bias for both a KDM5A module and a H3K4me3 module to be preferentially expressed in tissues where *KDM5A* gene is overexpressed. The difference in preferential expression of the H3K4me3 module in leukemia cells compared to normal blood cells suggests that this gene set may be deficient in methylation due to a distinct *HDM*/*HMT* gene expression signature, which includes a lack of correlation between *KDM5A* and *MLL1* expression. Furthermore, the KDM5A module displays strong anti-correlative behavior with an EZH2 module. The overall conclusion from this study is that the epigenome can be reduced to a combination of single gene modules, whose expression is a readout of *HDM*/*HMT* gene expression signatures.

Despite of remarkable progress in cancer research, one of the long-standing questions in the field is correlation between chromatin modifications and disease. While several studies have shown associations between a chromatin modifying enzyme, its representative target genes and patient outcome [24], there have been no studies linking multiple enzymes. However, sufficient evidence suggests that *cis*-interactions are common in chromatin-mediated transcriptional regulation. H3S10 phosphorylation enhances H3K4 methylation and H3K14 acetylation, and inhibits methylation at H3K9, thus facilitating chromatin decondensation [25]. Analysis of PcG and TrxG interactions and identification of bivalent marks encouraged the notion that there is a crosstalk between placing H3K4 methylation and H3K27 methylation at the same gene. The global analysis conducted here revealed a strong correlation in expression of genes displaying H3K4 methylation and genes displaying H3K27 methylation, suggesting that histone modifying activities are coordinated not only in *cis*- but also in *trans*- configuration. While our study does not offer precise mechanistic concepts to explain this coordination, we show that it depends on *HDM*/*HMT* gene expression signature, which can be subsequently tested using genetic approaches. We suggest that the *HDM*/*HMT* gene expression signatures result in maintaining transcriptional equilibriums at H3K4 and H3K27 methylated genes pertinent to a particular fate or to a diseased condition (Figure 6). Consistent with the paradigm proposed by Peter Nowell that neoplasia arises from cells with a growth advantage due to acquired genetic or epigenetic changes [26], a fundamental feature of cells populating the tumor may be a changed pattern of epigenetic modifiers, which bring a cell into a metastable state. Very little is known what causes the change in activity of HDM/HMT. Gain of function mutation of EZH2 and deletion of a microRNA, a negative regulator of EZH2 expression, have been linked to lymphomas and prostate cancer [27], [28]. While change in expression of single histone modifying enzymes has been linked to cancer, this study represents to our knowledge the first comprehensive analysis in cancer cell lines. Grouping cancer samples based on *HDM*/*HMT* gene expression signature, it should be possible to define new combinations of *HDM*s and *HMT*s that have not been considered yet to act directly in regulating gene modules associated with specific cancers and to develop novel markers to cancer subtypes.

Association of clinical predictions with genome chromatin structure was first appreciated in 2005, when Seligson and colleagues demonstrated that global levels of H3K4me2 and H3K4me3 are predictors of prostate carcinoma recurrence in patients with low-grade tumors [29]. We noticed multiple cases when the difference in expression shown in our heat map correlated with previously reported data on deregulation of the gene. Strikingly, *MLL1*, a frequent leukemia translocation partner, displays an expression pattern similar to *KDM5A*, whose translocation was also shown to be involved in leukemia [4]. Consistent with overexpression of a H3K36me2-specific demethylase KDM2B in T-cell and B-cell acute lymphoblastic leukemia and in acute myeloid leukemia [30], we found its overexpression in almost all hematopoietic cell lines. This increase may have functional significance, as ectopic expression of KDM2B is sufficient to transform hematopoietic progenitors while its depletion impairs HOXA9/MEIS1-induced leukemic transformation [31]. While our observation that cancer cells displayed changes in the level of multiple histone-modifying enzymes rather than changes in a particular enzyme seems frustrating at first sight, the observed highly significant correlations in the levels of different enzymes gives a promise that targeting one enzyme will be sufficient to change cancer cell properties. For example, it was shown that depletion of EZH2 by RNAi in prostate cancer cells diminished their tumorigenic and metastatic potential in vitro and in an orthotopic model in nude mice [32]. Strikingly, we found clusters of lung cancer samples that are more similar in *HDM*/*HMT* gene expression signature to hematopoietic malignancies (cluster 1 in Figure 3A) than to other clusters of lung cancer samples, which are more similar to colon and breast cancer samples (cluster 3 in Figure 3A). Because the *HDM*/*HMT* gene expression signature is similar in some cell types, there is a potential to use in these cells cancer therapeutics against the same target. It may be also possible to improve disease prediction algorithms as an Oncomine Molecular Concept Map (MCM) study showed that H3K27-methylated genes are downregulated in lung cancers and the lung gene expression signature was able to successfully predict the clinical outcome of prostate patients [33].

In contrast to genetic lesions, epigenetic alterations are potentially reversible, thus holding promise for therapeutic intervention. “Erasing” this state through inhibitors targeting DNA methyltransferases and histone deacetylases (HDACs) has been a widely used treatment for certain hematopoietic cancers and is being tested in clinical trials for other types of cancer. KDM5A is increased in drug-resistant cancer cell populations and its association with HDACs may underlie cell sensitivity to the HDAC inhibitors TSA and SAHA [21]. The involvement of KDM5A in transcriptional activation may be similar to the active role played by HDACs at the transcribed regions [34]. Thus, a combination of HDAC inhibitors and KDM5A inhibitors may be preferable for treatment of cancer cells with KDM5A overexpression. The homeotic gene *BRD8*, which reflects KDM5A activity, would be useful for testing the effects of KDM5A inhibitors. It is unclear if other KDM5 family members can be found at active genes. The KDM5B enables to catalyze demethylation of the same mark as KDM5A, however, it appears to have different expression pattern and therefore serve specific requirements in particular tissues.

While our results are consistent with previous findings on up- and downregulation of single enzymes, they represent an initial step to decipher enzyme combinations that can be co-regulated in cancer. Our data suggest that, in the absence of appropriate inhibitors to a causative HMT or HDM, pharmacological targeting of co-regulated *HDM*/*HMT* in the same gene expression signature could be applied to selectively target tumor cells. Interestingly, we used ChIP-on-chip, ChIPseq and gene expression data from non-matching cell types and still revealed correlations. This unexpected result suggests that it wouldn't be too challenging a task to conduct a gene expression analysis of the cancer epigenome since genome-wide data from different studies can be used. However, we anticipate that the outcome of correlation analyses will depend on whether the samples are from a more differentiated tissue or have stem cell properties. Because the likelihood of detecting a gene expression signature increases with the number of samples, creating profiles for a particular tumor type using large datasets will achieve more precision.

## Materials and Methods

Normalized mRNA expression data (Affymetrix Human U133A/GNF1H GeneAtlas microarray chip) [35] from 73 normal human tissues were downloaded from the BioGPS database [36]. Raw mRNA microarray expression data from various cancer cell lines (the GSK cancer cell line genomic profiling performed on Affymetrix GeneChip® U133 Plus 2.0 array) were downloaded from the National Cancer Institute's caBIG/caArray databases (https://cabig.nci.nih.gov/). Three replicates were available for each cell line. The mean expression was taken and intensity values were transformed to log_2_ values. When more than one probe was present for the same gene, the mean value was determined. However, in some cases, probes that did not have a good correlation of expression with respect to other probes of the same gene, or probes that did not express across any of the samples, were not included. Log_2_-converted mean absolute expression values were represented in a color-coded heatmap (Figure 1) using the program Matrix2Png (version 1.2, May 2010) [37]. For the GeneAtlas and GSK data in Figure 2 and 3, expression in each sample is presented in comparison with the mean value. In particular, normalized gene expression values were generated by subtraction of the gene's mean value followed by division by the standard deviation, and were delineated in a color-coded heatmap using the program Genesis [38]. Unsupervised hierarchical clustering of samples was performed using the Manhattan distance. The detailed probe level log_2_ expression data used in this study are presented in Table S2. Detailed normalized gene expression clustered data for cancer cell lines are presented in Table S6.

Target genes of KDM5A in U937 cell line (0 hr) were obtained from our previous study [5]. EZH2 targets in human ES cells were collected from the study by Ku and colleagues [19] and the genomic locations of enriched histone-binding sites (H3K4me3 and H3K27me3) were obtained from the study by Joseph and colleagues (GSE23701) [15]. Enriched peaks were annotated to the closest EnsEMBL gene transcription start site (version 54) [39] using the Bioconductor package ChIPpeakAnno [40]. Based on the “gene-normalized” expression of these targets in GeneAtlas normal cell data and GSK cancer cell line data, we carried out *z*-score median enrichment analysis using Gitools (www.gitools.org [41]), which compares the median expression value of the genes in each module (KDM5A and EZH2) with a distribution of random modules of the same size drawn from the expression values for the sample. The result is a *z*-score, which is a measure of the difference between the observed median expression value compared with the expected value. Positive and negative *z*-scores indicate significantly higher or lower expression level of genes in the module under this condition, respectively.

Pearson's correlation coefficient (PCC) of HDM and HMT expression profiles in normal tissues and cancer cell lines was analyzed using Gitools.

We used the TissueScan Cancer Survey Tissue qPCR panel 384-1 (OriGene, Cat# CSRT102), which contains 381 human tumor tissues. Some of the samples are normal non-malignant tissue samples, making it possible to compare expression in tumor versus normal tissue (Table S10). Samples with irregular melt peaks were removed, as this indicates non-specific amplification. Samples that failed to produce any product or samples with Ct values greater than 35 were assigned a value of 40. The baseline threshold values were set manually for each plate to ensure it was constant for the primer. Fold change was calculated using the 2^−ΔΔCt^ method relative to the mean ΔCt of each gene across all the samples in the array and normalized to the *B2M* gene. The expression level was therefore arbitrary but allowed us to see in which tissues expression was high or low relative to the mean value. Real-time PCR experiments were carried out using the CFX96 system and SsoFast EvaGreen Supermix (Bio-Rad). Each PCR contained 8.5 ng cDNA and 0.5 µM each primer: *KDM5A*, 5′-AAGAGAAGAAGATGACGATGATGAC-3′ and 5′-CCTGAAGCCTGGTGTCTGG-3′; *KDM5B*, 5′-AAGAGTAGCATCAAGCAAGAACC-3′ and 5′-GACATACAGGTCCACAGCATTG-3′; and *BRD8*, 5′-CATACTGTGACTGTTTCC-3′ and 5′-CAGCAAGGACAGCATCTTCTC-3′. The *B2M* primers have been described elsewhere [5]. The expression level of each gene in each tissue was represented as heatmaps produced with Gitools.

## Supporting Information

Figure S1 **Expression levels of** ***KDM5A*** **and** ***KDM5B*** **in human tumors (TissueScan Array).** Gene expression data are presented as in Figure 5, but with sample annotation. Samples are arranged according to tumor grade.
(TIF)

Figure S2 **Expression levels of** ***KDM5A*** **and** ***BRD8*** **in human tumors (TissueScan Array).** Gene expression data are presented as in Figure 5, with sample annotation. Samples are arranged according to tumor grade.
(TIF)

Table S1 **Human histone demethylases and histone methyltransferases.**
(DOC)

Table S2 **Absolute (log_2_) expression values of HDMs and HMTs in normal human tissues (GeneAtlas).** Datasets used in Figure 2(A) are highlighted with the corresponding colors.
(XLS)

Table S3 ***Z*** **-score enrichment analysis statistics for expression of target genes of KDM5A, EZH2 and genes displaying H3K4me3 or H3K27me3 in normal human tissues (GeneAtlas).**
(XLS)

Table S4 **Correlation coefficient of expression values of** ***KDM5A*** **,** ***EZH2*** **, KDM5A and EZH2 target genes, and genomic regions displaying H3K4me3 and H3K27me3 in normal human tissues (GeneAtlas).**
(XLS)

Table S5 **Correlation coefficient of expression values of** ***KDM5A*** **and H3K4 HMTs in normal human tissues (GeneAtlas).**
(XLS)

Table S6 **Normalized gene expression values (log_2_) of** ***HDM*** **and** ***HMT*** **in human cancer cell lines (GSK database).**
(XLS)

Table S7 ***Z*** **-score enrichment analysis statistics for** ***KDM5A*** **,** ***EZH2*** **and their target gene expression in human cancer cell lines (GSK database).**
(XLS)

Table S8 **Correlation coefficient of expression values of** ***KDM5A*** **,** ***EZH2*** **, KDM5A and EZH2 target genes, and genomic regions displaying H3K4me3 and H3K27me3 in human cancer cell lines (GSK database).**
(XLS)

Table S9 **Correlation coefficient of** ***HDM*** **and** ***HMT*** **expression values in human cancer cell lines (GSK database).**
(XLS)

Table S10 **RT-qPCR data for** ***KDM5A*** **,** ***KDM5B*** **and** ***BRD8*** **(TissueScan Array).** Expression values were calculated using the 2^–ΔΔCt^ method relative to the reference gene *B2M.* Sample annotation was provided by OriGene.
(XLS)
