# Supplementary figures and images for: Co-Regulation of Histone-Modifying Enzymes in Cancer
Source: PLoS One. 2011 Aug 23;6(8):e24023. doi: 10.1371/journal.pone.0024023 (PMC3160334; doi:10.1371/journal.pone.0024023)

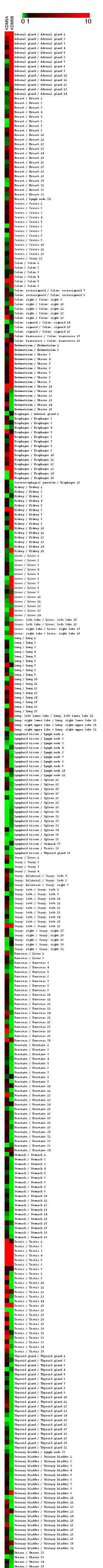

Supplement: Figure S1 — Expression levels of KDM5A and KDM5B in human tumors (TissueScan Array). Gene expression data are presented as in Figure 5, but with sample annotation. Samples are arranged according to tumor grade. (TIF) [file pone.0024023.s001.tif]

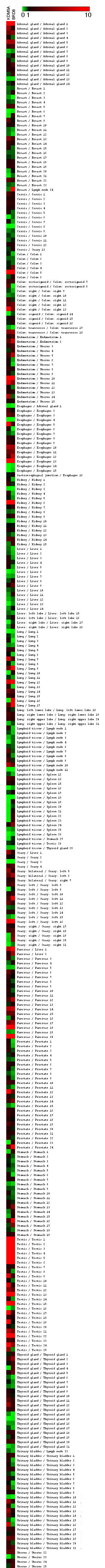

Supplement: Figure S2 — Expression levels of KDM5A and BRD8 in human tumors (TissueScan Array). Gene expression data are presented as in Figure 5, with sample annotation. Samples are arranged according to tumor grade. (TIF) [file pone.0024023.s002.tif]
