# Supplementary material for: Co-Regulation of Histone-Modifying Enzymes in Cancer
Source: PLoS One. 2011 Aug 23;6(8):e24023. doi: 10.1371/journal.pone.0024023 (PMC3160334; doi:10.1371/journal.pone.0024023)
Supplement: Table S1 — Human histone demethylases and histone methyltransferases. (DOC) [file pone.0024023.s003.doc]

**Table S1:** Human Histone Demethylases and Histone Methyltransferases

| **Gene name** | **Refseq ID** | **Superfamily** | **Substrate specificity** |
| --- | --- | --- | --- |
| KDM1A/LSD1 | NP_055828 | LSD1 | H3K4me2/me1 |
| KDM1B/LSD2 | NP_694587 | LSD1 | H3K4me2/me1 |
| JARID2 | NP_004964 | jmjC |  |
| JMJD6 | NP_055982 | jmjC | H3R2me2/H4R3me2 |
| KDM2A/JHDM1A/NDY2/FBXL11 | NP_036440 | jmjC | H3K4me3;H3K36me2/me1 |
| KDM2B/JHDM1B/NDY1/FBXL10 | NP_115979 | jmjC | H3K36me2/me1 |
| KDM3A/JHDM2A/JMJD1A | NP_060903 | jmjC | H3K9me2/me1 |
| KDM3B/JHDM2B/5qNCA | NP_057688 | jmjC | H3K9me2/me1 |
| KDM3C/JHDM2C/TRIP8 | NP_004232 | jmjC |  |
| KDM3D | NP_005135 | jmjC |  |
| KDM4A/JHDM3/JMJD2A | NP_055478 | jmjC | H3K9me3/me2;H3K36me3/me2 |
| KDM4B/JMJD2B | NP_055830 | jmjC | H3K9me3/me2;H3K36me3/me2 |
| KDM4C/JMJD2C/GASC1 | NP_055876 | jmjC | H3K9me3/me2;H3K36me3/me2 |
| KDM4D/JMJD2D | NP_060509 | jmjC | H3K9me3/me2;H3K36me3/me2 |
| KDM5A/JARID1A/RBP2 | NP_005047 | jmjC | H3K4me3/me2 |
| KDM5B/JARID1B/PLU1 | NP_006609 | jmjC | H3K4me3/me2 |
| KDM5C/JARID1C/SMCX | NP_004178 | jmjC | H3K4me3/me2 |
| KDM5D/JARID1D/SMCY | NP_004644 | jmjC | H3K4me3/me2 |
| KDM6A/UTX | NP_066963 | jmjC | H3K27me3/me2/me1 |
| KDM6B/JMJD3 | XP_043272 | jmjC | H3K27me3/me2/me1 |
| KDM6C/UTY | NP_009056 | jmjC | H3K27me3/me2/me1 |
| KDM7A/PKDM10A/JHDM1D | NP_085150 | jmjC |  |
| KDM7B/PKDM10B/PHF8 | NP_055922 | jmjC | H3K9me1/me2;H3K27me2;H3K36me2 |
| PKDM10C/PHF2 | NP_005383 | jmjC |  |
| PKDM11/JMJD4 | NP_075383 | jmjC |  |
| PKDM12A/JMJD5 | NP_079049 | jmjC |  |
| MLL1/MLL/HRX/KMT2A | NP_005924 | SET | H3K4 |
| MLL2/KMT2B | NP_003473 | SET | H3K4 |
| MLL3/KMT2C | NP_733751 | SET | H3K4 |
| MLL4/KMT2D | NP_055542 | SET | H3K4 |
| MLL5/KMT2E | NP_061152  NP_891847 | SET | H3K4 |
| SETD1A/SET1A/KMT2F/KIAA0339 | NP_055527 | SET | H3K4 |
| SETD1B/SET1B/KMT2G | NP_055863 | SET | H3K4 |
| SET7/SET9/KMT7 | NP_085151 | SET | H3K4 |
| ASH1/KMT2H | NP_060959 | SET | H3K4 |
| PRDM9 | NP_064612 | SET | H3K4 |
| SMYD3 | NP_073580 | SET | H3K4 |
| EZH2/KMT6 | NP_004447  NP_694543 | SET | H3K27 |
| SUV39H1/KMT1A | NM_003173 | SET | H3K9 |
| SUV39H2/KMT1B | NM_024670 | SET | H3K9 |
| G9a/KMT1C | NP_006700  NP_079532 | SET | H3K9 |
| EuHMTASE/GLP/KMT1D | NP_001138999  NP_079033 | SET | H3K9 |
| ESET/SETDB1/KMT1E | NP_001138887  NP_036564 | SET | H3K9 |
| CLL8/KMT1F | NP_001153780  NP_114121 | SET | H3K9 |
| RIZ1/KMT8 | NP_036363  NP_056950  NP_001007258  NP_001129082 | SET | H3K9 |
| PR-SET7-8/KMT5A | NP_065115 | SET | H3K20 |
| SUV4-20H1/KMT5B | NP_060105  NP_057112 | SET | H3K20 |
| SUV4-20H2/KMT5C | NP_116090 | SET | H3K20 |
| SET2/KMT3A | NP_054878 | SET | H3K36 |
| NSD1/KMT3B | NP_758859  NP_071900 | SET | H3K36 |
| SMYD2/KMT3C | NP_064582 | SET | H3K36 |
| DOT1L/KMT4 | NP_115871 |  | H3K79 |
